# Social evaluation and imitation of prosocial and antisocial agents in infants, children, and adults

**DOI:** 10.1371/journal.pone.0235595

**Published:** 2020-09-16

**Authors:** Elena Vaporova, Norbert Zmyj

**Affiliations:** Institute of Psychology, TU Dortmund University, Dortmund, Germany; Jiangsu Normal University, CHINA

## Abstract

The question of whether infants prefer prosocial agents over antisocial agents is contentious. Therefore, the first goal of the present study was to replicate previous findings regarding infants’ preference. The second goal was to assess whether infants are more likely to imitate a prosocial agent than an antisocial agent. We tested 9-month-old, 14-month-old, and 4-year-old children. The study used the “opening a box to get a toy” paradigm in which an animal puppet is trying unsuccessfully to open a box and is either helped by a prosocial puppet or hindered by an antisocial puppet. We presented these social events via video, and subsequently administered an imitation task. As an additional control, adults were asked to describe the videos showing the prosocial and antisocial agent. Although most adults were able to identify both agents, the three age groups of children did not prefer the prosocial agent over the antisocial agent, and were not more likely to imitate the prosocial agent. The lack of differences might be explained by methodological issues or by a lack of robustness of the effect.

## Introduction

It is essential for humans to know who will help and who will hinder them in achieving their personal goals in future interactions. One way to evaluate another person’s inclination to help or to hinder goal achievement is to extrapolate from that person’s past behavior: Somebody who helps another individual will probably be helpful in the future, and somebody who harms another individual will probably be harmful in the future. During the first 18 months of life, infants become sensitive to other people’s need for help [1–3] and to other people’s cooperative or competitive behavior towards the infants themselves [4]. However, the question of whether infants are also able to evaluate another person’s prosociality if they observe the person in interaction with others remains unresolved.

Recently, a growing body of research has targeted infants’ ability to evaluate others’ prosocial and antisocial behavior. This line of work rests on the infants’ ability to understand intentions which has been documented in several studies (e.g., [4–6]). In an initial study, Hamlin, Wynn, and Bloom [7] found that 6- and 10-month-old infants prefer prosocial agents over antisocial agents. The authors presented infants with a social scenario, called the “climbing a hill” paradigm. In this paradigm, a wooden block with glued-on eyes is struggling to climb a hill and is either helped by a prosocial agent or hindered by an antisocial agent. When given the choice between the two agents, infants preferred the prosocial agent. These findings were replicated by Hamlin, Wynn, and Bloom [8] with 3-month-old infants. Moreover, they were further replicated by Hamlin and Wynn [9] using two new paradigms and different age groups: Infants aged 3, 5 and 9 months were presented with two different paradigms. The first paradigm was the “opening a box to get a toy” paradigm, in which an animal puppet tries but fails to open a box to get a rattle and is either helped by a prosocial puppet or hindered by an antisocial puppet. The second paradigm was the “retrieving a dropped ball” paradigm, in which an animal puppet accidentally drops its ball and is again either helped by a prosocial puppet or hindered by an antisocial puppet. Studies by Hamlin and colleagues [7, 8, 10] showed that infants as young as 3 months of age preferred prosocial agents, as expressed by looking longer at them and that infants as young as 5 months of age preferred prosocial agents, as expressed by reaching for them, which has been the common measure to test infants’ preferences [11]. To explain these results, Hamlin [12] argued that infants have an innate moral core, meaning that “morality is a core aspect of human nature” (p. 191).

A number of studies have tried to replicate and extend the findings of Hamlin and colleagues [7, 8, 10]. The results were often in line with the original findings (e.g., [13–24]) with seven studies not conducted by Hamlin and colleagues [13, 14, 19–22]. This overall impression was confirmed by a recent meta-analysis that focused on young children’s manual choice between a prosocial and an antisocial agent [11]. However, this meta-analysis revealed a potential publication bias towards reporting statistically significant results and a potential effect of the research group in which a study was conducted. The meta-analysis also found evidence that the infants’ and young children’s preference for prosocial over antisocial others depends on the type of scenario (e.g. giving and taking away vs. helping to achieve a goal and hindering to achieve a goal). Accordingly, the general proclivity of infants and young children to prefer prosocial agents might also depend on other factors that we have not fully understood and that might lead to failed replications.

One line of debate concerned the “climbing a hill” paradigm. In a replication study by Scarf, Imuta, Colombo, and Hayne [25] with 10-month-old infants, the authors did find a preference for the prosocial agent. They suggested that this preference might be an artifact because in the original studies by Hamlin and colleagues the climber bounced up and down only on helping trials, but not on hindering trials. In Scarf et al.’s study this variable was manipulated so that the climber bounced up and down either on helping trials, or on hindering trials, or on both trials. As the infants’ preference was predicted by the bouncing event, the authors concluded that the underlying motivation of infants’ preferences in this paradigm is based on their preference for agents who bring about effects and not on their preference for prosocial agents. In response to this criticism, Hamlin [17] showed that Scarf et al. [25] themselves introduced new elements which might have obscured infants’ preference for prosocial agents (e.g., an odd gaze direction of the climber).

Other replication studies ranged from exact replications that followed the original procedure as closely as possible to conceptual replications that picked up the general idea of infants’ preference for prosocial agents but changed essential parts of the original studies. Many replication studies changed some details (e.g., presentation mode or participants’ age) which makes it difficult to conclude whether null results represent a failed replication. Two replication attempts that used the “climbing a hill” paradigm did not find a preference for the prosocial agent in infants [26] or in children [27]. Another two replication attempts used the original methods of the “opening a box to get a toy” paradigm, but failed to find any preference for the prosocial agent in 9-month-old infants [28] and in different age groups ranging from 9-month-old infants to 5-year-old children [29]. Another replication study exposed 19- and 32-month-old infants to the “retrieving a dropped ball” paradigm using colored cartoons which resulted in a successful replication [30]. A second study [31] tested 6-, 12-, and 18-month-old infants in three different social scenarios (help, play or share) using an eye-tracking methodology, but found mixed results: Six-month-olds preferred the prosocial character in the play scenario but the antisocial character in the help scenario, while 12-month-olds showed a preference for the prosocial character only in the help scenario; 18-month-olds showed no preference at all. Salvadori et al. [28] who did not replicate the original findings speculated that “[t]his pattern raises the possibility of a ‘lab effect’, i.e. a tendency for one team of researchers to find consistently stronger effects when using a given experimental paradigm” (p. 8). This speculation was confirmed by the recent meta-analysis regarding infants’ preference for prosocial agents [11]. In sum, the evidence of infants’ preference for prosocial over antisocial behavior is inconsistent. Further research from different research groups is needed to clarify the robustness of a preference for prosocial agents in infancy.

In real life, there are multiple instances in which infants could prefer prosocial over antisocial agents. However, this issue remains almost uncharted territory because infants’ preference for prosocial agents is usually investigated via reaching behavior. A fruitful extension of this line of work is to investigate a potential proclivity to learn from prosocial agents in infancy. Infants’ imitative behavior [32] is considered to be a central component of their learning of novel skills and behaviors. In fact, one-year-olds learn one to two new behaviors each day via imitation [33]. However, infants do not imitate automatically every action they have observed. Infants imitate rather selectively on the basis of certain characteristics of an agent. For example, 14-month-old infants were more likely to imitate novel actions when they were performed by an agent who had previously acted competently and confidently compared to when they were performed by an agent who had previously acted incompetently and unconfidently [34]. This selectivity might be driven by infants’ motivation to learn novel actions that are effective to achieve a goal. However, imitation also represents a way to communicate nonverbally with others or to identify with others [35]. This social side of imitation was demonstrated in a study in which an agent presented an action in a social manner (i.e., speaking to the infant before performing the action) and in a neutral manner (i.e., not soliciting the infant’s attention before performing the action). Infants were more likely to imitate actions presented in a social manner than in a neutral manner [36].

A previous study has reported that an agent’s prosociality influences infants’ imitation. In this study, a prosocial agent preferred one type of food whereas an antisocial agent preferred another type of food [37]. Infants were more likely to adopt the food preference of the prosocial agent than that of the antisocial agent. It is evident that infants had learnt about the agents’ preferences. However, human’s capacity to imitate does not only consist of reproducing the same end state (e.g., copying food choice), but also to copy the specific actions leading to this end state [38, 39]. Accordingly, established imitation tasks do not only focus on achieving the end state of an action but also on the action itself [39, 40]. Therefore, the question arises of how robust this finding is and whether it still holds when an imitation task includes copying end states as well as actions. There is ample evidence that even in the first year of life, infants are already able to imitate a three-step action that is directed to a hand puppet resembling a mouse (e.g. [40]). In this task, the agent grasps a mitten that is located on the paw of a hand puppet resembling a mouse, shakes it, and then puts it back on the hand puppet. Infants were found to be more likely to perform this action when the agent had performed the demonstration compared to a condition in which the infants had not previously observed the demonstration [40]. Recently, a cross-cultural study showed that a model’s previous prosocial or antisocial behavior did not influence 4-8-year-olds’ imitation of multi-step actions [41]. This null result in older children highlights the importance of using more sophisticated actions in this line of research.

Therefore, the present study pursued two goals. The first goal was to replicate the findings of Hamlin and Wynn [9] using the “opening a box to get a toy” paradigm. Due to the mixed findings in infants, we conducted three experiments, also testing older children and adults, to ensure that the task is well understood. Infants’ preference for prosocial agents over antisocial agents has been demonstrated when the scenario was presented in real-life [9] as well as on video [42]. Since these paradigms might be sensitive to minor changes in demonstration [25], we decided to present participants with video demonstrations in order to keep the demonstration constant across conditions. In previous studies, the presentation mode (real-life vs. televised) did not influence the infants preference for the prosocial agent [11]. Infants’ and children’s preference was assessed by their manual choice between the prosocial and antisocial agent which is a common measure in research field [11]. Adults were asked to describe the scenario.

The second goal of the present study was to assess whether infants are more likely to imitate the prosocial agent than the antisocial agent. Following [40], we used an elicited imitation task to test whether infant and children are more likely to imitate a prosocial agent than an antisocial agent.

Based on Hamlin and Wynn [9, 37], we predicted that children aged 9, 14, and 48 months would show a preference for the prosocial agent and would be more likely to imitate a prosocial agent than an antisocial agent. Adults were expected to demonstrate their differentiation between the prosocial and antisocial agent in their verbal report.

## Experiment 1

The first experiment was conducted with the 9- and 14-month-old infants in order to replicate the previous findings of Hamlin and Wynn [9] and to examine whether infants’ imitation is influenced by prosociality.

### Method

#### Participants

Participants were 55 healthy full-term infants (26 boys, 29 girls) aged 9 months (*n* = 25, *M* = 9 months; 5 days, *SD* = 12 days) and 14 months (*n* = 30, *M* = 14 months; 2 days, *SD* = 12 days). A further six additional infants participated but were excluded due to fussiness (*n* = 2), failure to choose a puppet in the social evaluation task and unwillingness to touch the puppet in the imitation task (*n =* 1) or procedural error (*n =* 3). In the social evaluation task, six out of the 55 children (one 9-month-old and five 14-month-olds) were excluded because they did not choose a puppet. Infants and their parents were recruited from a database of parents who had previously agreed to participate in child development studies. Written consent was obtained from the parents of the infants. At the end of their visit, infants received a small gift and a €5 expense allowance. The experiment was approved by the ethics committee for psychology of the Ruhr-Universität Bochum (no. 278).

#### Design

This study consisted of one social evaluation task, followed by one imitation task. The social evaluation task used a within-subject design. Each child witnessed one prosocial and one antisocial agent acting in a social event. The imitation task used a between-subject design. Infants witnessed either the prosocial or the antisocial agent performing a novel three-step action.

#### Materials

In the social evaluation task, a transparent plastic box (28 x 24 x 8 cm) containing a rattle (16 x 20 x 6 cm), two hand puppets resembling cows, one wearing a blue and the other wearing a yellow t-shirt (18 x 12 x 24 cm, henceforth labeled “cow puppets”), and one hand puppet resembling a girl (12 x 10 x 22 cm, henceforth labeled “protagonist”) were used. The social events were shown on a 20-inch monitor (Dell, UltraSharp 2007FBp, screen resolution 1600 x 1200). A camera that was positioned directly above the monitor recorded the infants’ face.

Eight video sequences were created by counterbalancing the t-shirt color (blue, yellow), the side of the prosocial and the antisocial hand puppet (left, right), and the social event itself (prosocial, antisocial). Two of these video sequences (e.g., S1 Video: the agent with the blue t-shirt was located on the left side and performed the prosocial act, S2 Video: the agent with the yellow t-shirt was located on the right side and performed the antisocial act), were used for testing one participant. In the imitation task, a grey hand puppet resembling a mouse (height = 30 cm) was used. The mouse hand puppet wore a mitten on its right paw. During the video demonstration, the mitten contained a bell. A second mitten without the bell was used when the mouse hand puppet was handed to the child (as in [40]). Two videos were created with either the blue or the yellow cow hand puppet acting on the mouse hand puppet. Only one video was used for testing one participant (e.g., the blue cow hand puppet performed the target actions).

#### Procedure

**Fig 1:**
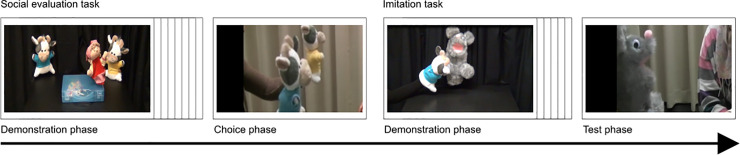
Still frames of the video sequences/video recordings in the different phases.

Infants and their parents were first escorted to the laboratory. The infants were allowed to explore the room and to get familiar to the female experimenter while parents were introduced to the experimental procedure and filled out the informed consent. In the testing area of the laboratory, the infants sat on their parents’ lap at a table approximately 80 cm away from the monitor. All sessions were videotaped. The procedure consisted of two tasks: A social evaluation task followed by an imitation task (see Fig 1 for the order of tasks and phases).

*Social evaluation task*. In the social evaluation task, infants were first presented with the transparent box containing the rattle to familiarize the infants to the box. Parents were instructed not to interact with the infants during the experiment. To attract the infants’ attention, the video showed a smiling sun and a bell rang before each video sequence started. The social evaluation task comprised a demonstration phase and a choice phase.

**Fig 2:**
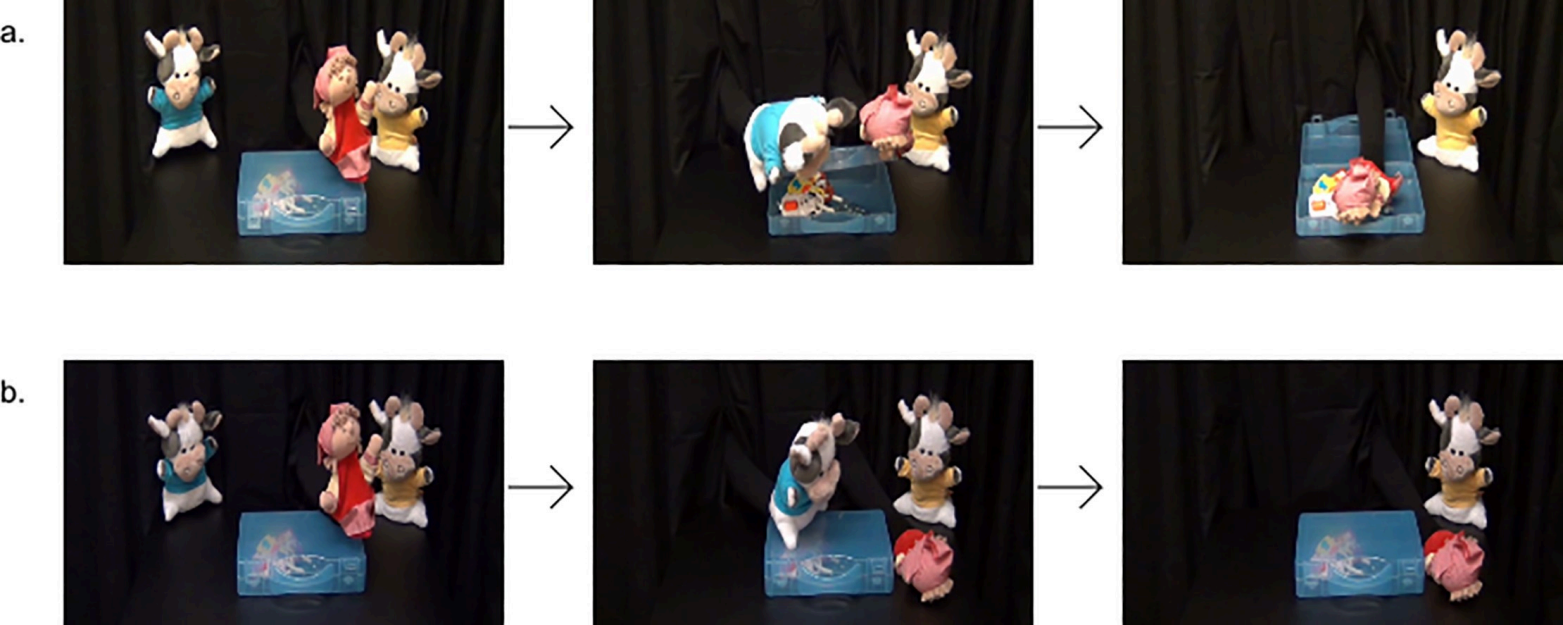
Screenshots taken from video showing (a) a sequence of a prosocial event and (b) a sequence of an antisocial event.

The demonstration phase consisted of eight trials. Each trial started with the protagonist moving to one side of the box, leaning over the box, and looking twice at the rattle inside. The protagonist then jumped onto the box, grabbed the lid, and attempted to open it five times. During the fifth attempt, one of the cow puppets entered from the back, helping or hindering the protagonist. In the prosocial trials, one of the cow puppets (i.e., the helper) grabbed the lid on the other side of the box, and the two agents (i.e., helper and protagonist) opened the box together (see Fig 2). Afterwards, the helper ran off stage and the protagonist sank into the box, grabbing the rattle. In the antisocial trials, one of the cow puppets (i.e., the hinderer) jumped onto the box and slammed it shut. While the hinderer ran off stage, the protagonist dropped down next to the box, without the rattle (see Fig 2 and supporting information).The four prosocial and the four antisocial trials were presented in an alternating order and the order of events (prosocial trial first, antisocial trial first) was counterbalanced across infants. Each trial lasted approximately 22 s. The infants saw the trials in a consecutive order. The color of the helper’s and the hinderer’s t-shirts, and the side on which the agent was shown (from the participant’s perspective) were counterbalanced across infants. The t-shirt color remained the same for the helper and for the hinderer within one infant.

In the choice phase, parents were instructed to turn their chair 90 degree to the right. The experimenter took a seat in front of the infant, presenting the prosocial and the antisocial puppet while saying, “Look who I’ve got here!” As soon as the infant had looked at both puppets, the experimenter put both puppets within reach (30 cm) of the infant saying, “Which one do you like?”

*Imitation task*. The social evaluation task was followed by the imitation task for which the parent and the infant returned to their initial position in front of the monitor. The task consisted of a demonstration phase and a test phase. In the demonstration phase, infants saw one of the cow hand puppets that were introduced in the social evaluation task. The cow hand puppet performed a three-step action: (1) pulling the mitten from the paw, (2) shaking the mitten three times up and down and (3) putting the mitten back onto the paw. Due to a video deficit in infants until the age of three [40, 43, 44], the video was presented six times.

In the test phase, infants received the mouse hand puppet with a mitten that produced no sound when shaken to ensure that infants were not triggered shaking the mitten when they accidently heard the bell ringing (see [40]). Parents were instructed to move their chair by approximately 1 m away from the screen and not to touch the mouse hand puppet. The experimenter took a seat in front of the infant, putting the mouse hand puppet within reach of the infant and saying, “Now you may play with the mouse!” After the infants had touched the mouse hand puppet, they had 90 s to reproduce the target actions.

#### Coding

In the social evaluation task, infants’ looking time during the demonstration phase was measured via Interact (Mangold). In the choice phase, infants’ choice was defined as the first puppet they touched while looking at it.

In the imitation task, it was coded which of the three target actions infants performed. “Pulling the mitten from the paw” was coded for any behavior which led to the removal of the mitten. “Shaking the mitten” was coded if infants shook the mitten up and down at least once. “Putting the mitten back onto the paw” was coded if infants touched the right paw with the mitten. A sum imitation score from 0 to 3 was calculated, with one point awarded for each target action that was reproduced by the infant. A second observer rated the behavior to assess interrater reliability (Cohen’s κ = .79).

### Results and discussion

The infants watched the presentation for most of its duration (*M* = 90.90%, *SD* = 8.05). There was no significant difference between the amount of time spent looking at the antisocial agent (*M* = 91.53%, *SD* = 8.72) and the prosocial agent (*M* = 90.26%, *SD* = 9.91, *t*(54) = 0.99, *p* = .325).

#### Social evaluation task

**Fig 3:**
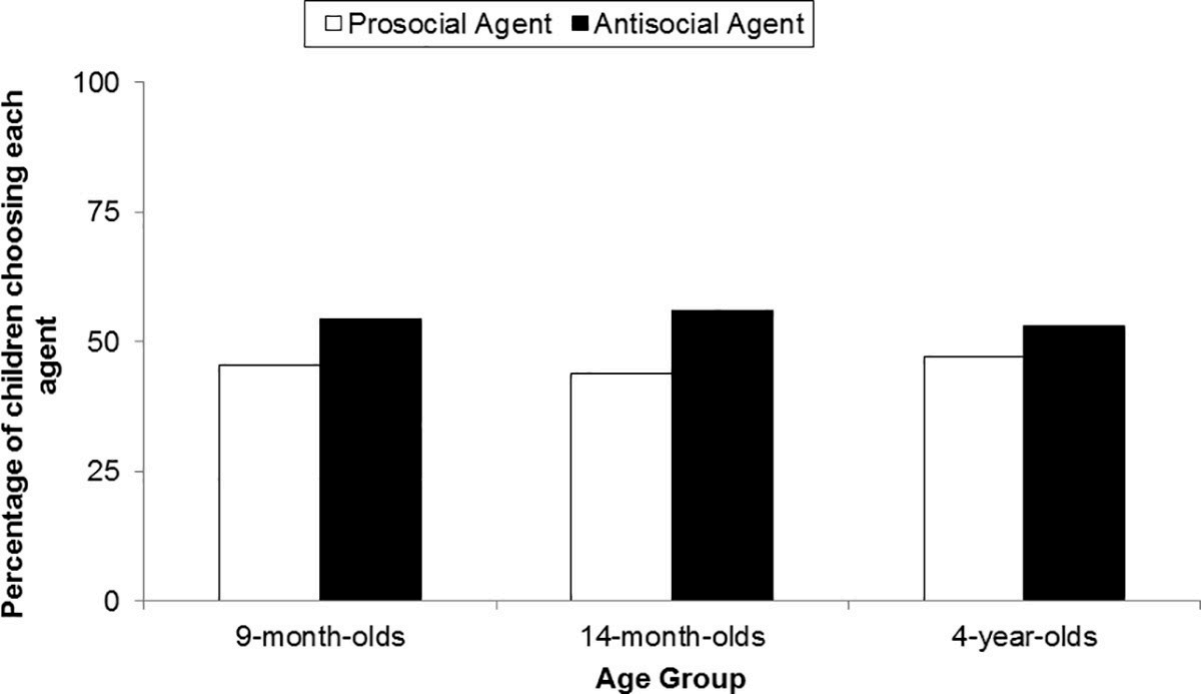
Percentages of infants (Experiment 1) and children (Experiment 2) who chose the prosocial and the antisocial agent.

Fig 3 shows the percentages of infants who chose the prosocial and the antisocial agent, respectively. The infants did not show a preference for the prosocial agent (23 out of 52, *p* = .488, binomial test). The results indicated no significant difference in the preference for the prosocial agent between the two age groups, *χ^2^*(1) = 0.01, *p* = .920.

Across both age groups, there was no effect of order of events and color of t-shirt of the prosocial agent on the infants’ choice (Fisher’s exact test, both *p*s > .05). However, an effect of the side of the prosocial agent did emerge (Fisher’s exact test, *p* < .001): Sixteen infants (30.8%) chose the puppet to their left whereas 36 infants (69.2%) chose the puppet to their right (*p <* .*008*, binomial test).

#### Imitation task

**Fig 4:**
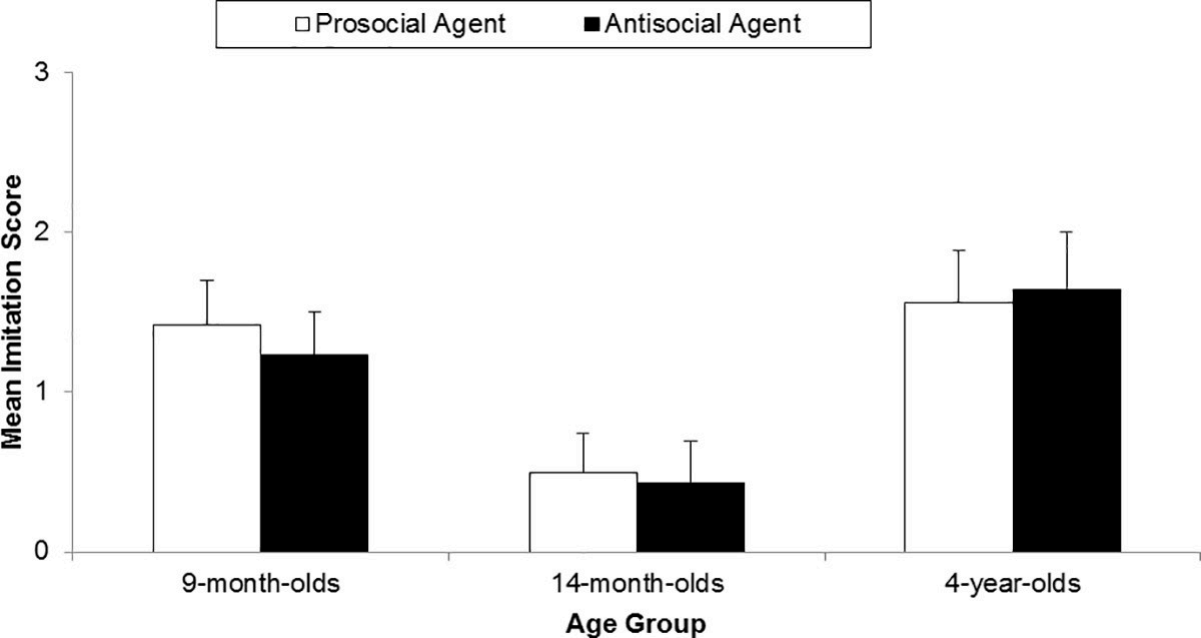
Mean imitation score for the prosocial and the antisocial agent in infants (Experiment 1) and children (Experiment 2). Error bars indicate standard errors.

Fig 4 shows the mean imitation rate of the prosocial and antisocial agent in 9- and 14-month-old infants. A two-way ANOVA was conducted to test the effect of age group (9-month-olds, 14 month-olds) and the effect of the cow puppet’s prosociality (prosocial, antisocial) on the imitation score, as well as the interaction between age and prosociality. No interaction effects between age group and the social identity of the agent emerged, *F*(1, 51) = 0.05, *p* = .830. There was also no main effect of the social identity of the agent, *F*(1, 51) = 0.24, *p* = .629. However, we found a significant main effect of age group, *F*(1, 51) = 10.53, *p* = .002, indicating that in general, the 9-month-old infants had higher imitation scores (*M* = 1.32, *SD* = 1.07) than the 14-month-olds (*M* = 0.86, *SD* = 0.47).

To explore whether the group of infants who chose the prosocial agent (*n* = 21) showed more imitation when watching the prosocial agent compared to when watching the antisocial agent, we conducted a Mann-Whitney *U* test. There was no difference in imitation scores between the two groups, *U* = 36.50, *Z* = -0.70, *p* = .519.

## Experiment 2

In Experiment 2, we tested whether 4-year-old children showed the expected preference for the prosocial over the antisocial agent in the choice test event (as in [30]) as well as increased imitation after observing the prosocial agent compared to the antisocial agent. To test whether the prosocial and the antisocial intention were salient, we also asked the children about what had happened in the video sequences. We tested 4-year-old children because previous research revealed that children in this age group have a core sense of morality and are able to verbally express this capacity (e.g., [45–48]).

### Method

#### Participants

Participants were 34 healthy full-term children (22 boys, 12 girls) aged 4 years (*M* = 4 years; 7 months; 5 days, *SD* = 3 months; 27 days). In the social evaluation task, two out of the 34 children were excluded because they did not choose a puppet. In the imitation task, seven out of the 34 children were excluded due to unwillingness to touch the puppet. Children and their parents were recruited from local kindergartens and a database of parents who had previously agreed to participate in child development studies. Written consent was obtained from the children’s parents. At the end of the experiment, children received a small gift, and if they were tested at the university, parents received a 5 € expense allowance. The experiment was approved by the ethics committee for psychology of the Ruhr-Universität Bochum (no. 278, amendment).

#### Design

The experimental design was identical to that in Experiment 1. Additionally, a short interview was conducted after the choice test event.

#### Materials

The materials were identical to the materials in Experiment 1, except for the monitor on which the video stimuli were presented (Apple, MacBook Air, screen resolution 1440 x 900).

#### Procedure

Testing took place in three German kindergartens (*n* = 15) or the university laboratory (*n* = 19). During the entire session, children sat on a chair suitable for children. Each child was tested separately and each session was videotaped.

*Social evaluation task*. As in Experiment 1, the social evaluation task consisted of a demonstration phase and a choice phase. The procedure of the demonstration phase was identical to that in Experiment 1 except that only 6 social events (3 prosocial, 3 antisocial) were presented, because piloting revealed that children became easily bored with 8 social events.

After the demonstration phase, children’s chairs were rotated 90° to the right. In the choice phase, the experimenter took a seat in front of the child, presented the prosocial and antisocial puppets and said, “Look who I’ve got here! Which one do you like best?”

*Interview*. Immediately after the choice test event, the experimenter told the child that she would like to ask some questions. Question 1 was “Which cow helped the girl in the video?”, and Question 2 was “What happened in the video?” Due to a potential carry-over effect from the first question to the second question, we dropped the second question from the analysis.

*Imitation task*. The procedure was the same as in Experiment 1.

#### Coding

In the social evaluation task, a choice of the child was coded when he or she named the color of or pointed to the puppet that he or she liked best. In the interview, question 1 was coded as “correct” if the child named the helper and as “incorrect” in all other cases. The coding for the imitation task was similar to Experiment 1. A second rater coded 30% of the videos; interrater reliability lay at kappa = 1.

### Results and discussion

#### Social evaluation task

The children did not show a preference for the prosocial agent (15 out of 32, *p* = .860, binomial test). Forty-seven percent of children chose the prosocial agent and 53% chose the antisocial agent (see Fig 3). There was no effect of order of events, color of t-shirt and side of the prosocial agent on the children’s choice (Fisher’s Exact test, all *p*s > .05).

#### Interview

Seventeen of the children who chose one of the puppets in the choice task identified the helper correctly (53.1%), and the remaining 15 children did not identify the helper correctly (46.9%). There was no significant difference between the amount of correct identification and the amount of incorrect identification, *χ*^2^(1, *N* = 32) = 0.125, *p* = .724. Correctly identifying the helper did not covary with children’s preference for the helper, *χ^2^*(1) = 2.08, *p* = .149.

#### Imitation task and explorative analysis

There were no significant differences between the imitation score of the prosocial (*M* = 1.56, *SD* = 1.32) and antisocial agent (*M* = 1.64, *SD* = 1.21, *U* = 86.50, *Z* = 0.08, *p* = .942, Mann-Whitney *U*-Test) as depicted in Fig 4.

We additionally explored whether the group of children who chose the prosocial agent (*n* = 12) showed more imitation when watching the prosocial agent compared to when watching the antisocial agent. No significant difference in imitation scores emerged between the groups, with children imitating the prosocial agent (*N* = 7, *M* = 1.57, *SD* = 1.51) not more frequently than the antisocial agent (*N* = 5, *M* = 1.40, *SD* = 1.34, *U* = 15.50, *Z* = -0.35, *p* = .755, Mann-Whitney *U*-Test).

Neither infants (Experiment 1) nor children (Experiment 2) preferred the prosocial agent over the antisocial agent. This failure to find a preference for the prosocial over the antisocial agent in the choice task and in the imitation task is in line with previous non-replications of the original studies [26–29] but in contrast to other studies that reported a differentiation between prosocial and antisocial agent in young children (e.g., [49]). To test whether the intentions of the helper and the hinderer were sufficiently salient, we conducted a third experiment, in which we tested adults.

## Experiment 3

To test whether the attribution of prosocial and antisocial intentions to the agents is intuitive, we conducted a third experiment. In Experiment 3, we asked adults to describe the demonstration videos which were presented to the children in Experiments 1 and 2.

### Method

#### Participants

Participants were 26 undergraduate students (6 male, 20 female) with a mean age of 24.31 years (range = 18–39). All were students of the local university, provided written informed consent, and received course credit for their participation. The experiment was approved by the ethics committee for psychology of the Ruhr-Universität Bochum (no. 278, amendment).

#### Design

The study used a within-subject design. Each participant watched the same videos of the prosocial and antisocial event as the participants in Experiments 1 and 2.

#### Materials

The materials were identical to those used in Experiment 1.

#### Procedure and coding

Participants were told that they were going to watch two short video sequences three times in succession and that they would be asked to describe them afterwards. The order of events, color of the puppets’ t-shirt and side of the agents were counterbalanced. After watching the videos, the participants received the following instruction: “Please describe in one sentence what you saw in the videos”, in order to examine how well the adults understood the social scenarios. Participants’ judgments were coded as “identified both correctly” if they used words like “prosocial”, “helper” or “helping” to describe the helping scenario and words like “antisocial”/”not prosocial”, “hinderer”, “hindering”/”not helping” to describe the hindering scenario. If they did not use any of the appropriate words to describe the hindering scenario, but did use the correct words to describe the helping scenario, their judgments were coded as “identified the helper correctly”. Again, a second coder coded 30% of the descriptions. The interrater reliability was kappa = 1.

## Results and discussion

The descriptions of the social scenarios showed that the majority of the adults (72%) identified both the helper and the hinderer correctly. The remaining 28% identified the helper correctly. Accordingly, the social event we used appears to at least be understandable for adults. We did not ask the adults for their preferences. In a previous study [27], the adults’ looking times were measured and they did not prefer a helper or hinderer scenario in the “climbing a hill” paradigm.

## General discussion

The goal of the present study was to replicate the findings of Hamlin and Wynn [9] and to assess whether children imitate a prosocial agent more likely than an antisocial agent. Although our procedure was analogous to Hamlin and Wynn’s [9] procedure, none of the three groups of infants and children preferred the prosocial agent or were more likely to imitate the prosocial agent than the antisocial agent. Whereas Hamlin and colleagues (for an overview, see [12]) replicated the original findings, which was confirmed by a recent meta-analysis [11], other studies [26–29, 31, 50] have failed to replicate them. Our findings add to these non-replications, as we found that infants and children do not prefer a prosocial agent over an antisocial agent and that they are not more likely to imitate a prosocial agent than an antisocial agent.

An explanation for the lack of effect might lie in minor methodological differences between the original study by Hamlin and Wynn [9] and the present study. Such differences might be especially relevant for this type of task. For instance, Steckler, Woo, and Hamlin [42] highlighted the importance of details of the testing environment when testing infants such as the color of the curtains. The following differences were apparent in our study: First, we presented the participants with different hand puppets compared to Hamlin and Wynn [9]. While the latter authors used hand puppets resembling grey and orange cats, our hand puppets resembled cows, and wore yellow or blue t-shirts. Infants and children may have focused on the similarities between the cows and less on the color of the t-shirt, according to which the agent could be identified as prosocial or antisocial. Second, our videos in the demonstration phase had a slightly longer duration than the original version (22 s vs. 15 s). Accordingly, in our study, infants might have lost interest more easily than in the original study. However, the infants followed the demonstration for most of the time (i.e., around 90% proportional looking time). Third, we used a familiarization paradigm, while the original study [9] used a habituation paradigm. We used a familiarization paradigm, because we intended to keep our study analogous to the previous study on imitating prosocial agents [37]. Habituating infants to a visual display sometimes distresses infants. Since there was an additional imitation task after the social evaluation task, we opted for a familiarization paradigm because infants are usually more focused afterwards. Although we used a similar amount of demonstration videos (8 trials in the present study, 9 trials in [9]) not all infants might have comprehended the scenario because they might have needed more demonstrations. Fourth, we presented the social events on video, instead of presenting them as a real-life puppet show. On the one hand, other studies have demonstrated infants’ differentiation between prosocial and antisocial agents using video presentations (e.g., [42, 51]). As other studies using the original methods [28, 29], also failed to replicate the original findings, it is doubtful whether these differences can explain the lack of effects found in the present study. The issue of presentation mode was investigated in a meta-analysis and the results revealed that the presentation mode (real-life vs. televised, real-life vs. cartoon) did not influence the infants’ preference [11]. On the other hand, there is evidence of a video deficit effect [52], which describes young children’s poorer performances in tasks using video demonstrations compared to real-life demonstrations. This might explain why some studies failed to replicate the original effect (e.g., [31] that used an animated cartoon), although it cannot explain why other studies using real-life agents did not replicate the original effect (e.g., [28, 29]).

One successful replication study by Scola and colleagues [30] found a significant preference for prosocial agents using the “retrieving a dropped ball” paradigm. The “opening a box to get a toy” paradigm, as used by Maxwell and Raftseder [29], Salavadori and colleagues [28] and in the present study, might be susceptible to minor changes in the procedure when studying preference for prosocial agents. One line of future studies on infants’ preference for prosocial agents might be to investigate whether the type of paradigm or which type of cues in the “opening a box to get a toy” paradigm influences infants’ preference for prosocial agents.

In our experiments, infants were more likely to choose the prosocial agent when it was shown on the right-hand side from the infants’ perspective. This side bias might reflect infants’ development of right-handedness [53, 54] and could obscure a possible preference for the prosocial agent. Following the idea of Holvoet et al. [31] and Hinten et al. [27], future studies could use an eye-tracking methodology to study infants’ preferences in order to bypass their developing handedness.

When replicating original findings, sample size is an important issue. In Hamlin and Wynn’s [9] original study an effect size of 75% (Cohen’s *g* = 0.25) was reported. The optimal sample size for a replication study is *N* = 49 (alpha error = .05, power = .95, two-tailed) which is in line with the sample size of Experiment 1 (*N* = 55). In contrast, a recent meta-analysis [11] revealed an effect size of 68% (Cohen’s *g* = 0.18). Under this condition, the optimal sample size would be *N* = 99. Accordingly, the sample size in Experiment 1 might have been too small to replicate the effect of the agent’s prosociality. However, this meta-analysis included infant studies with different paradigms. Since the meta-analysis found considerable variation of results depending on the paradigm, it is not the best frame of reference for calculating sample sizes.

The absence of a preference for the prosocial agent over the antisocial agent in 4-year-old children was surprising. Previous research that presented children with pro- and antisocial agents showed that 4-year-olds have a sense of good and bad [55–57]. Likewise, toddlers also differentiated between prosocial and antisocial agents [10]. Unlike typical infant studies, this study presented toddlers with verbal cues that further enriched the social event and might have made it easier for the toddlers to understand the protagonist’s intention. These verbal cues might be crucial when testing toddlers and children. Another way to interpret our null results in 4-year-olds is to question the quality of the video demonstrations. However, the video demonstrations were analogous to the video demonstrations that were used in previous studies using this paradigm (see supporting information). Also, Experiment 3 showed that adults do understand the content. An alternative way to interpret our results is that infants’ preference for prosocial agents is based on subtle cues that are not described in the studies with positive findings. Hence, these cues can be easily missed which results in failed replications. The evidence of potential effects of the research group or type of task [11] should be a further motivation to investigate the nature of failed replications.

Besides the failure to find a preference for prosocial agents, the infants and children in our study also did not imitate the prosocial agent more than the antisocial agent. This finding is inconsistent with the results of Hamlin and Wynn [37], who reported that 16-month-old infants imitated the food preference of a prosocial agent more than the food preference of an antisocial agent. However, the findings in 4-year-olds are consistent with another study that did not report an influence of a model’s prosociality or antisociality on 4-8-year-olds’ imitation [41].This may be attributable to the lack of a preference for the prosocial agent, as the imitation task was based on the assumption that infants and children show a preference for prosocial agents. As we did not find a preference for prosocial agents, it may be the case that the infants and children did not evaluate the antisocial agent as antisocial and therefore imitated both agents equally. However, a number of alternative explanations might also explain this null result. First, the lack of difference in the imitation scores could be the result of using a mitten that contained a bell in the demonstration phase and a mitten without a bell in the test phase. This slight change in the material might have reduced the continuity between both phases. We consider this explanation as unlikely because previous studies also used televised models with the identical material. These studies could elicit imitation in 6-18-month-old infants(e.g. [58]). Second, all previous studies using this imitation task showed a human model. In contrast, a hand puppet model was used in our imitation task. Since even minor differences in a model’s characteristics such as reliability [59] or age [60] changes the likelihood of infants’ imitation, using a hand puppet model could have increased the difficulty of the imitation task. One reason for this increased difficulty might be that the infants did not interpret the action of the hand puppet as intended. Previous research showed that infants re-enact failed actions performed by an adult but not by a mechanical device [61], presumably because they could infer the adult’s intention but not the intention of the mechanical device. Although infants have observed a complete action in the present study, they still might have had difficulties inferring the intention of hand puppet agent which could have resulted in a lower imitation rate. Third, the mean imitation score was also quite low in children. Since the demonstrated target actions were easy to remember, 4-year-olds might have been unsure what was expected from them.

To conclude, our study did not replicate the original findings of Hamlin and Wynn [9]. Our findings further highlight the fragile nature of infants’ preference for prosocial over antisocial agents. Future studies should aim to identify the reasons for the mixed empirical support for the assumption of an innate moral core in infancy.

## Supporting information

S1 Data (XLSX)

S1 Video Helper. (MPG)

S2 Video Hinderer. (MPG)
